# Cognitive Measures and Performance on the Antisaccade Eye Movement Task

**DOI:** 10.5334/joc.52

**Published:** 2019-01-24

**Authors:** B. B. Magnusdottir, E. Faiola, C. Harms, E. Sigurdsson, U. Ettinger, H. M. Haraldsson

**Affiliations:** 1 Landspitali University Hospital, Department of Psychiatry, Reykjavik, IC; 2 Reykjavik University, Department of Psychology, Reykjavik, IC; 3 University of Bonn, Department of Psychology, Bonn, DE; 4 University of Iceland, School of Health Sciences, Reykjavik, IC

**Keywords:** eye movements, working memory, attention, cognitive control

## Abstract

The antisaccade (AS) task is considered a prominent measure of inhibitory control, but it is still unclear which cognitive processes are used for successful performance of the task. Previous results have provided evidence for the involvement of several processes, including working memory (WM), inhibition and attention. Thus, the aim of this study was to explore, using a range of neuropsychological tests, which cognitive factors predict individual differences in AS performance. To do so, 143 healthy participants underwent a battery including tests measuring inhibition, working memory, cognitive flexibility, sustained attention, IQ and fluency. Hierarchical stepwise regression analyses were conducted to assess the association with AS performance. Performance on the Trail-Making-Test, version B (TMT-B), a test measuring flexibility, divided attention and WM, was found to significantly predict AS latency. Rapid Visual Information Processing (RVIP), used to assess sustained attention and WM, significantly predicted AS error rate. Other cognitive measures, however, did not significantly predict AS performance. Bayesian Model Averaging supported these conclusions and showed that non-significant predictors are unlikely to be associated with AS outcomes. Several explanations are provided for the associations of TMT-B and RVIP with AS performance; as the tests measure a range of different cognitive processes, interpretation of these results remains less clear. For a better understanding of the cognitive mechanisms underlying AS performance, future research should make use of a wider range of attention and WM tests.

Despite a large number of studies using the antisaccade (AS) task to investigate cognitive function, researchers still debate which cognitive processes are used for performing the task. In the AS task, subjects are asked to fixate on a central dot and when a target stimulus appears either in a left or right peripheral location, the subject has to refrain from the dominant response of moving the eyes towards the stimulus (prosaccade) but instead has to move the eyes to the opposite peripheral location (antisaccade) (Hallett, 1978). The AS task has four main measures; AS gain, AS latency, AS spatial error and AS error rate of reflexive saccade errors. AS error rate reflects the percentage of error trials over the total number of valid trials. This is considered a measure of inhibitory control and the ability to appropriately activate a volitional response (McDowell & Clementz, 2001; Reuter, Rakusan, & Kathmanna, 2005). AS latency is defined as the time from target appearance to saccade initiation and reflects the speed of volitional response generation (Reuter & Kathmann, 2004). AS gain and spatial error are measures of the ability to match saccade amplitude to target amplitude which depends on sensorimotor processes involved in transforming the covertly encoded visual target location into a motor output (Moon et al., 2007).

In the healthy population, the mean error rate is around 20% (Hutton & Ettinger, 2006), but significantly more errors are made by people with severe mental illness such as schizophrenia (Hutton & Ettinger, 2006) and people with frontal lobe lesions (Guitton, Buchtel, & Douglas, 1985). Imaging studies demonstrate the importance of fronto-parieto-subcortical networks, including frontal eye fields (Connolly, Goodale, Goltz, & Munoz, 2005; Connolly, Goodale, Menon, & Munoz, 2002; Ettinger et al., 2008; McDowell et al., 2005; Pierrot-Deseilligny, Ploner, MüRi, Gaymard, & Rivaud-PéChoux, 2002; Ploner, Gaymard, Rivaud-Péchoux, & Pierrot-Deseilligny, 2005), dorsolateral prefrontal cortex (McDowell et al., 2005; Pierrot-Deseilligny et al., 2002), and its connections to subcortical areas (Ploner et al., 2005). Performance on the AS task has been linked to the control of purposeful behavior and there is evidence for the involvement of several cognitive processes, such as inhibition, working memory and attention (Hutton, 2008).

Here, our aim was to explore the association of different cognitive processes with AS task performance in an individual differences study and to relate the findings to earlier frameworks as well as more recent experimental studies on this association in order to contribute to current literature on cognitive processes underlying successful AS performance (Hutton, 2008; Massen, 2004; Munoz & Everling, 2004).

Inhibition processes have been suggested to be important for successful AS performance as the novel target is thought to evoke automatic processes, i.e. the prosaccade, that has to be inhibited for the AS response to be evoked (Everling & Fischer, 1998; Guitton et al., 1985; Roberts, Hager, & Heron, 1994). However, the role of a dedicated inhibition or stop process in successful AS response has been questioned (Cutsuridis, Smyrnis, Evdokimidis, & Perantonis, 2007; Hutton, 2008; Hutton & Ettinger, 2006; Massen, 2004). Instead, parallel processing of an internally triggered AS and an automatic saccade response to the target has been proposed; if the AS response is processed fast enough, the saccade response to the target is cancelled. Relatedly, it has been suggested that one of the roles of the prefrontal cortex is goal activation and maintenance, and that inhibitory control may emerge as a consequence of that but not as a separate function (Munakata et al., 2011). Development of the prefrontal cortex of monkeys is in line with this notion: the ability to inhibit behavioral responses is thought to improve with age as a result of better ability to form an alternative plan (and not due to improved ability to suppress the impact of a prepotent stimulus) (Zhou et al., 2016).

Studies using task manipulations support the importance of attention for successful performance on the AS task. In a study where a cue was presented in the peripheral location opposite to the subsequently presented target, performance was worse despite the cue being presented in the direction of a correct AS (Weber, Dürr, & Fischer, 1998). The authors suggested that instead of providing a cue towards the correct response the cue provided a shift in attention and triggered an AS response as the participants were instructed to move their eyes in the opposite direction of the stimulus, as in a typical AS task (Weber et al., 1998). In the same line, Kristjansson (2007) put forward the competition model of AS generation. The model has been supported by a series of studies indicating that by adding an attentionally demanding task (Kristjansson, 2001) or a somatosensory stimulus (Kristjansson, 2004), that both slow down the prosaccade response, the AS response can be speeded up (Kristjansson, Chen, & Nakayama, 2001; Kristjansson, Vandenbroucke, & Driver, 2004).

Several studies have investigated the association between performance on cognitive tests and the AS task in healthy participants but the results are mixed. Significant associations have been reported between AS error rate and (a) the Stroop interference score (Aichert et al., 2012; Levy, Mendell, & Holzman, 2004), and (b) perseverative errors during the Wisconsin Card Sorting Test (WCST) (Crawford, Bennett, Lekwuwa, Shaunak, & Deakin, 2002; Radant, Claypoole, Wingerson, Cowley, & Roy-Byrne, 1997), although others have not found an association with WCST (Barton et al., 2002; Snitz, Curtis, Zald, Katsanis, & Iacono, 1999) and generally correlations with other cognitive performance measures tend to be low (Aichert et al., 2012; Miyake et al., 2000; Rey-Mermet, Gade, & Oberauer, 2018; Stahl et al., 2014).

The positive associations of Stroop and WCST with AS performance have been interpreted as an indication of the importance of frontal lobe function (Radant et al., 1997) and more specifically to support the importance of inhibition processes for successful AS performance (Crawford et al., 2002). Others, however, have pointed out that inhibition processes only explain a small portion of the variance of AS performance (Aichert et al., 2012), and have suggested that other factors are also important for successful AS performance (Levy et al., 2004). Specifically, the association between tests of working memory (WM) and AS performance has been investigated in several studies on healthy participants. Studies on verbal WM capacity have indicated associations with performance on the AS task (Mitchell, Macrae, & Gilchrist, 2002; Roberts et al., 1994; Unsworth, Schrock, & Engle, 2004). The positive associations of verbal WM and AS performance have been related to the effects of increased WM demand on AS performance and interpreted as the interference with the goal to make antisaccades (Kane, Bleckley, Conway, & Engle, 2001). It has been suggested that WM capacity is in part an attentional construct (Chuderski, 2014; Meier, Smeekens, Silvia, Kwapil, & Kane, 2017; Shipstead, Harrison, & Engle, 2015), with the dual-component model of WM capacity indicating that both attention control and memory based abilities might be important for WM (Unsworth & Engle, 2007; Unsworth & Spillers, 2010).

In summary, theories and experimental studies of the cognitive mechanisms underlying AS performance support models of parallel processing of prosaccade and antisaccade movements where the two processes compete, contrary to models emphasizing specific inhibition or stop processes. Results from studies on the association of performance on cognitive tests with AS performance in the healthy population are mixed, and in many of the studies cited above only the associations of one or few cognitive factors with AS performance were tested in each study, mostly in relatively small samples, and often only in student samples. Research with a broader cognitive battery and a larger group of participants from the general population is therefore needed.

With the aim of extending our understanding of the cognitive mechanisms underlying successful AS performance, the purpose of the current study was to explore which cognitive processes best predict successful AS response. A large group of healthy subjects from the general population performed both the AS task and a cognitive test battery measuring WM, response inhibition, flexibility and sustained attention as well as fluency, and IQ as a measure of general cognitive ability.

## Methods

### Subjects

This study is a part of a larger investigation of genetic effects on cognition in psychiatric and neurological disorders (see e.g. Stefansson et al., 2014). Results from the control group were used for analysis in the present study. All subjects had been genetically screened and had no large copy-number variants (CNVs) that have been associated with schizophrenia and autism, nor history of neurological or severe psychiatric illnesses, eye diseases, severe head injury or substance abuse/dependence in the past 12 months. Participants (N = 143) were recruited from the local community through a study recruitment center in Reykjavik and all were Icelandic and between 18 and 65 years old (see Table 1). Approval for the study was obtained from the Icelandic Scientific Ethics Committee and The Data Protection Authority. Following a full description of the study to participants, written consent was obtained. Further information on the selection of participants can be found in Stefansson et al. (2014).

**Table 1:** Descriptive statistics of variables used in the regression analysis.

N	143
Age (Mean/SD)	42.51 (10.06)
Females/Males (N)	78/65
IQ (Mean/SD)	106.25 (14.37)
TMT-B^1^ (Mean/SD)	62.59 (21.95)
WCST^2^ (Mean/SD)	9.87 (8.39)
Stroop (Mean/SD)	24.42 (8.72)
LFL^3^ (Mean/SD)	15.50 (4.74)
CFL^4^ (Mean/SD)	23.92 (5.82)
SWM^5^ (Mean/SD)	13.49 (12.01)
RVIP^6^ (Mean/SD)	0.92 (0.05)
AS^7^ Gain (Mean/SD)	–110.66 (28.76)
AS Latency (Mean/SD)	291.01 (49.16)
AS Spatial Error (Mean/SD)	41.73 (16.60)
AS Error Rate (Mean/SD)	35.57 (20.25)

*Notes:* ^1^Trail Making Task Version B, ^2^Wisconsin Card Sorting Task, ^3^Letter Fluency Task, ^4^Category Fluency Task, ^5^Spatial Working Memory Task, ^6^Rapid Visual Information Task, ^7^Antisaccade.

### Cognitive Tasks

Cognition was assessed with the following tests and below is a description of the specific test measurements used for the analysis in this study.

**Stroop.** The Stroop task is considered a measure of inhibition (Miyake et al., 2000; Stroop, 1935). An Icelandic paper and pencil version was used, derived from the Golden version (Golden & Freshwater, 1978). It includes three sections, all of which contain words or color pads placed in 10 rows and 5 lines placed on a A4 sheet. On the first sheet, called Stroop I, the time it takes to read one page of color names written in black ink is measured, in Stroop II the time it takes to name the colors of color pads is assessed, and finally in Stroop III the subjects are required to name the color of a word that is actually the name of another color. A derived score aimed at controlling for both speed of reading and color naming (Stroop III – ((Stroop II + Stroop I)/2)) was used for analysis as it is thought to be a good measure of inhibition (Scarpina & Tagini, 2017; Van der Elst, Van Boxtel, Van Breukelen, & Jolles, 2006).

**WCST.** The Wisconsin Card Sorting Test (WCST) was designed to measure abstract reasoning and the ability to shift cognitive strategies in response to environmental cues (Berg, 1948) and has been used as an indication of inhibition of mental set (i.e. Crawford et al., 2002). The standard computerized version with 128 cards was administered (Heaton & Staff, 1993). Ratio of perseverative errors was used for the analysis, reflecting the errors made when a subject persists in responding according to an older rule after the rule has been changed.

**Spatial Working Memory.** The Spatial Working Memory (SWM) subtest from the computerized CANTAB battery was used. The task requires retention and manipulation of visuospatial information (Feigenbaum, Polkey, & Morris, 1996; Owen, Downes, Sahakian, Polkey, & Robbins, 1990). In the test, participants were told to search for a blue token that was hidden beneath one of eight boxes on the screen and that the token would be hidden at a different place each time so they should not look under a box where the token had already been hidden. The measure used for the analysis was the count of times the subject revisited one of the 8 boxes where a token had previously been found, called between search errors for 8 boxes, as there were 8 boxes on the screen during this trial.

**TMT-B.** A paper and pencil version of the Trail Making Test B (TMT-B) was administered (Reitan, 1958; Reitan & Wolfson, 1985), measuring the time it took to draw a line when shifting between numbers (1–13) and letters (A-L) in ascending order according to guidelines by Strauss et al. (2006). The TMT-B is thought to measure flexibility, divided attention (Lezak et al., 2012; Lezak, Howieson, & Loring, 2012) and working memory (Sánchez-Cubillo et al., 2009).

**RVIP.** The Rapid Visual Information Processing (RVIP), also a subtest from the CANTAB battery, is thought to assess the ability to sustain attention over a period of time (Sahakian & Owen, 1992) as well as WM (Coull, Frith, Frackowiak, & Grasby, 1996). In the task, digits from 2 to 9 are presented in a pseudo-random order and participants are asked to press a button as fast as they can when one of the sequences 2-4-6, 3-5-7 or 4-6-8 appear on the screen. The variable used for the analysis was A’, which is a measure of sensitivity to errors regardless of error tendency. It measures the ability to detect target sequences by using probability of hits, which is the proportion of correct responses given when a target sequence is presented on the screen, and probability of false alarms, the proportion of responses when the target sequences are not presented.

**Letter Fluency.** Letter fluency was tested with the Controlled Oral Word Association Test (COWAT) (Benton, 1989). It was measured with two trials, in each of which subjects had one minute to name as many words as possible that begin with the letters H and then the letter S, in the Icelandic translation. The mean number of words starting with the two letters was calculated and used for the analysis (letter fluency (LFL)).

**Category Fluency.** Category fluency (CFL) was measured with one trial with the number of animals named in one minute.

**WASI.** The Wechsler Abbreviated Scale of Intelligence (WASI), first edition, was administered as an indication of intelligence (IQ estimation) and used as a control variable for the regression analysis. A standardized Icelandic version of the WASI was recently published (Gudmundsson, 2016), and a trial version of that publication was used in this study. Therefore, the IQ estimation score was calculated using the control group of the current study as local norms for different age groups (see further description of calculation in Stefansson et al., 2014).

### Antisaccade Task

Antisaccades were measured using an IRIS eye tracker, model 6500 (Skalar Medical BV, Delft, The Netherlands). As only conjugate eye movements over a relatively short amplitude range were made, only left eye movements were recorded. The visual stimulation programs for eye movement testing were run on a computer with a 16-inch screen and were purpose written in C++ for Windows (Psyal Ltd., London, UK). The visual target was a white circular dot (subtending about 0.3° of visual angle) presented on a black background, with 17 cm from the eyes to the centre of the screen. In each trial, the target was first in the central location (0°) for 1000–2000 ms and then stepped to one of four peripheral locations (±6°, ±12°) where it remained for 1000 ms. Participants were instructed to look at the target while in the central position and redirect their gaze to the exact mirror image location of the target as soon as it moved to the side. Each peripheral location was used 15 times, resulting in a total of 60 trials.

AS eye movements were analyzed with a semiautomatic routine in the EYEMAP software (AMTech GmbH, Dossenheim, Germany). Saccades were detected on minimum amplitude (1°), velocity (30°/s) and latency (100 ms) criteria. AS performance was assessed using the following parameters: AS gain, AS latency, AS spatial error and AS error rate. *AS gain* (%) was calculated as the primary saccade amplitude divided by target amplitude multiplied by 100; *AS latency* of correct AS was defined as the time (ms) from target appearance to saccade initiation. *AS spatial error* (%) was obtained by calculating, for each saccade, the percentage of residual position error; and *AS error rate* (%) reflects the percentage of error trials over the total number of valid trials, excluding e.g. blink trials.

### Statistical Analysis

Statistical analyses were carried out using IBM SPSS Statistics 22.0 (IBM, Armonk, NY). Significance was set at *p* < 0.05. All variables were tested for normality of distribution, which was assumed when skewness was between –1 and +1. Variables that were not normally distributed were transformed using log transformation; specifically, variables from the TMT-B, WCST and Stroop tests met criteria for transformation. For each of the criterion variables (AS gain, AS latency, AS spatial error and AS error rate) hierarchical stepwise regression analyses were conducted. In addition to the performance variables of the different cognitive tasks (the predictors), control variables were included in the regression analyses. As some of the dependent variables correlated with age, sex and IQ, these three variables were chosen as control variables. The following variables were used as predictors: performance time (in seconds) in the TMT-B, number of perseverative errors in the WCST, the time for naming colors in the Stroop task (in seconds), the mean number of words in the LFL, the total number of words in the CFL, the between search errors in the SWM task with eight boxes (number of errors), as well as A’ in the RVIP. In the first step of the regression analyses, the three control variables entered into the regression model. The second step included the predictor variables using the stepwise method.

In addition to the stepwise regression approach, we used Bayesian statistics for further investigation. Mostly, we were interested in the role of non-significant predictors in the stepwise regression, i.e. whether the non-significance (and, thus, the non-inclusion in the model) was due to a null-effect or due to a Type-II error. While this question cannot easily be answered and Bayesian statistics is not concerned with error rates, it can provide quantified uncertainty to derive conclusions about the role of different predictors.

Specifically, we used Bayes factors and Bayesian Model Averaging (BMA; Hoeting et al., 1999, Raftery et al., 1997, Wang et al., 2004, Wasserman, 2000).^1^ BMA in particular provides information comparable to hierarchical stepwise regression, i.e. (i) the posterior probability for the best model and (ii) probabilities for each predictor being non-zero (inclusion probability). The latter can be used to derive conclusions about the relevance of a predictor for the four antisaccade outcomes. For BMA, competing candidate models are considered. For our analysis, the intercept term and the covariates age, sex, and IQ are included in all models (inclusion probability of 1). There are seven candidate predictors (as in the stepwise regression) and four different outcomes (as the four criterions in the stepwise regression). That is, there are 128 possible models for each outcome, which are included as candidate models in the model averaging. Since we chose not to include prior information in the analysis and did not expect any model to perform better than any other, all models had prior probabilities proportional to one (i.e. all models are *a priori* equally likely). For all regression coefficients, the Jeffreys-Zellner-Siow (JZS) prior (Liang et al., 2008) with a scale parameter of 1 was used as a default prior. Bayes factors reported for the models in the stepwise regression JZS-priors with a scale parameter of 1 were also used. Bayes factors between 1/3 and 3 are considered inconclusive and posterior inclusion probabilities below .50 represent no evidence for the predictor being relevant (Jeffreys, 1961).

The BMA analysis was performed using R 3.5.1 and the *BAS* package (version 1.5.1) by (Clyde, 2018). For the reported Bayes factors the *BayesFactor* package was used. The combined approach using Markov Chain Monte Carlo and Bayesian Adaptive Sampling was used for computing the model probabilities.

## Results

Descriptive statistics (means and standard deviations) of control variables, predictors and criterion variables can be found in Table 1. An overview of their correlations is shown in Table 2. Posterior model probabilities and inclusion probabilities for each predictor are shown in Table 3.

**Table 2:** Correlations between age, sex, cognitive and antisaccade measurements.

Variables		1		2		3		4		5		6		7		8		9		10		11		12		13	

1	Age																										
2	Sex	.14																									
3	IQ	–.03		–.22	**																						
4	TMT-B^1^	.19	*	–.12		–.50	**																				
5	WCST^2^	.07		.24	**	–.45	**	.33	**																		
6	Stroop	.22	*	–.06		–.31	**	.35	**	.08																	
7	LFL^3^	–.09		–.12		.49	**	–.44	**	–.22	*	–.28	**														
8	CFL^4^	–.11		–.12		.41	**	–.33	**	–.29	**	–.25	**	.54	**												
9	SWM^5^	.35	**	.27	**	–.33	**	.28	**	.30	**	.23	**	–.29	**	–.26	**										
10	RVIP^6^	–.15		.01		.38	**	–.43	**	–.28	**	–.44	**	.30	**	.27	**	–.31	**								
11	AS^7^ Gain	–.02		–.17	*	.01		–.01		–.02		–.08		.04		.01		–.04		.02							
12	AS Latency	.24	**	.09		–.16		.28	**	.20	*	.18	*	–.22	**	–.13		.24	**	–.15		.17	*				
13	AS Spatial Error	.18	*	.18	*	–.05		–.01		.03		.13		–.04		–.05		.10		–.09		–.69	**	–.14			
14	AS Error Rate	.09		.13		–.17	*	.15		.23	**	.19	*	–.13		–.08		.09		–.30	**	–.14		.16		.19	*

*Notes:* ^1^Trail Making Task Version B, ^2^Wisconsin Card Sorting Task, ^3^Letter Fluency Task, ^4^Category Fluency Task, ^5^Spatial Working Memory Task, ^6^Rapid Visual Information Task, ^7^Antisaccade, * *p* < .05, ** *p* < .01.

**Table 3:** Best models, posterior probabilities for the best models and inclusion probabilities for each predictor.

Dependent variable	Predictor	Best model	Pr(β ≠ 0|Y)	Posterior model probability of best model

AS^7^ Gain	Intercept	•	1.000	0.276
	Age	•	1.000	
	Sex	•	1.000	
	IQ	•	1.000	
	TMT-B^1^		0.176	
	WCST^2^		0.146	
	Stroop		0.284	
	LFL^3^		0.155	
	CFL^4^		0.146	
	SWM^5^		0.145	
	RVIP^6^		0.154	

AS Spatial Error	Intercept	•	1.000	0.284
	Age	•	1.000	
	Sex	•	1.000	
	IQ	•	1.000	
	TMT-B		0.164	
	WCST		0.151	
	Stroop		0.264	
	LFL		0.144	
	CFL		0.144	
	SWM		0.144	
	RVIP		0.185	

AS Latency	Intercept	•	1.000	0.188
	Age	•	1.000	
	Sex	•	1.000	
	IQ	•	1.000	
	TMT-B	•	0.725	
	WCST		0.265	
	Stroop		0.211	
	LFL		0.278	
	CFL		0.155	
	SWM		0.253	
	RVIP		0.157	

AS Error Rate	Intercept	•	1.000	0.249
	Age	•	1.000	
	Sex	•	1.000	
	IQ	•	1.000	
	TMT-B		0.160	
	WCST		0.351	
	Stroop		0.219	
	LFL		0.156	
	CFL		0.168	
	SWM		0.205	
	RVIP	•	0.934	

*Notes:* Bayesian Model Averaging was used to determine inclusion probabilities for the predictors. Thus, regression coefficient estimates are not included. For inference on effect sizes, the estimates for the stepwise regression are reported in the text. ^1^Trail Making Task Version B, ^2^Wisconsin Card Sorting Task, ^3^Letter Fluency Task, ^4^Category Fluency Task, ^5^Spatial Working Memory Task, ^6^Rapid Visual Information Task, ^7^Antisaccade.

In step one of the regression analysis for AS gain, the control variables entered the model, explaining 0.9% of the variance. The control variable sex showed a significant correlation with the criterion (β = –0.18, *p* = 0.041), although the model fit was not significant and statistical evidence was in favor of the null model (*p* = 0.233, BF_10_ = 0.103). None of the predictors correlated significantly with the criterion (all *p* > 0.176) and the Bayesian analysis showed posterior inclusion probabilities below .30 for all non-covariate predictors (see Table 3). This result shows that the variables not contained in the best model (first) very likely did not have an influence on AS gain.

For AS spatial error, the model with the control variables explained 3.5% of the variance and the model fit was significant, but the Bayes factor against the intercept-only model was inconclusive (*F*_(3,139)_ = 2.72, *p* = 0.047, BF_10_ = 0.517). However, none of the control variables correlated significantly with the criterion (all *p* > 0.063). Furthermore, there was no significant correlation between the predictors and the criterion (all *p* > 0.204). The Bayesian model averaging showed posterior inclusion probabilities again below .30 for all non-covariate predictors (see Table 3).

For AS latency, the first step of the regression analysis revealed that the control variables explained 5.9% (*R^2^_Adjusted_* = 0.059) of the variance. The control variable age correlated significantly with the criterion (β = 0.23, *p* = 0.006). The model fit of the first model was significant, but Bayes factor shows inconclusive evidence, although in favor of the covariate model against the null model (*F*_(3,139)_ = 3.96, *p* = 0.010, BF_10_ = 2.420). In the second step of the analysis, the TMT-B entered the model as a significant predictor for AS latency (β = 0.26, *p* = 0.009). The final model accounted for an increased amount of variance explained of 4.4% (Δ*R^2^* = 0.044), this change was significant (*p* = 0.009, BF_10_ = 6.390 comparing the new model against the covariate model). The total amount of variance explained was 9.7% (*R^2^_Adjusted_* = 0.097), and the final model was significant (*F*_(4,138)_ = 4.83, *p* = 0.001, BF_10_ = 15.464 comparing the new model against the null model containing only the intercept). Furthermore, the Bayesian model averaging results yielded a posterior inclusion probability of 0.725 for TMT-B. For all other predictors, inclusion probability was again below. 30.

For AS error rate, none of the control variables correlated significantly with the criterion (all *p* > 0.085), the model fit was not significant and statistical evidence was in favor of the null model (*F*_(3,139)_= 2.03, *p* = 0.112, BF_10_ = 0.216). However, the second step of the analysis showed that RVIP was a significant predictor of the AS error rate (β = –0.29, *p* = 0.001). The second model explained 8.5% of the variance (*R^2^_Adjusted_* = 0.085), corresponding to a change in *R^2^* of Δ*R^2^* = 0.069. The change (*p* = 0.001, BF_10_ = 30.230) and the final model was significant (*F*_(4,138)_ = 4.30, *p* = 0.003, BF_10_ = 4.210). Furthermore, the Bayesian model averaging results yielded a very high posterior inclusion probability of 0.934 for RVIP. For all other predictors, inclusion probability was below .30, apart from the WCST (Pr(β ≠ 0/Y) = .351) which still, however, yielded an inclusion probability below .50 and thus not indicating evidence in favor of a relevant association.

## Discussion

The aim of this study was to explore which cognitive processes best account for performance on the AS task. A large group of healthy participants from the general population was tested with a wide range of cognitive tests and the association with performance on the AS task was assessed with a series of hierarchical stepwise regression analyses followed up by Bayesian statistics. The results indicate that the time taken to complete the TMT-B significantly predicts AS latency, and RVIP performance is a significant predictor of error rate on the AS task. Other cognitive variables, however, did not significantly predict AS performance variables.

The results of the Bayesian analysis support the findings of the stepwise regression. RVIP was found to be a very good predictor of AS error rate and TMT-B a strong predictor of AS latency. In both cases, the model including the predictor was the best model with the highest posterior model probability. For both AS gain and spatial error, the null model containing only the intercept and covariate terms was the best model when compared to models including any other predictor. In addition, inclusion probability for all predictors was very low for these two dependent variables, which is in line with the regression analysis, where no predictor correlated significantly with the criteria.

The RVIP is a continuous performance task thought to measure sustained attention, which is the ability to maintain attention on a particular object or task over a period of time (Clyde, 2018). The test has frequently been used and interpreted as a relatively pure measure of attention (Gau & Huang, 2014; Pagnoni, 2012). However, research has indicated that verbal WM may be required for RVIP processing (Coull et al., 1996). It is therefore not entirely clear how to interpret the significant association found between performance on RVIP and error rate on the AS task in this study. It could be concluded that the ability to sustain attention is an important correlate of the ability to evoke correct AS response, but that verbal WM function similarly is an important predictor of error rate on the AS task. It has also been suggested that WM is in part an attentional construct (Chuderski, 2014; Kane et al., 2001; Meier et al., 2017; Shipstead et al., 2015) and therefore one possible explanation does not necessarily exclude the other.

The TMT-B is traditionally thought to be a measure of set shifting or flexibility, as well as divided attention (Lezak et al., 2012). The results of our study show that the time to complete the TMT-B predicts AS latency, but as the TMT-B is a measure of more than one cognitive process it is again not entirely clear how to interpret these results. It can be argued that adequate flexibility or the ability to shift between two mental sets is necessary for successful AS performance. The association could also indicate support for the importance of attention for successful AS performance, suggesting that such performance demands both selective attention (RVIP) and flexibility. On the other hand, a recent study on the construct validity of TMT-B found verbal WM to be the strongest predictor of TMT-B performance (Sánchez-Cubillo et al., 2009). Parallel processing models of AS eye movements have emphasized the role of WM, and earlier studies have found associations between verbal WM and AS performance (Mitchell et al., 2002; Roberts et al., 1994; Unsworth et al., 2004).

Importantly, the neuropsychological tests used in this study all measure more than one cognitive construct, a phenomenon known as the task impurity problem (Miyake et al., 2000). Therefore, the associations of RVIP and TMT-B with AS performance in this study are difficult to interpret, despite the specific cognitive process labels often attached to these tasks. As both RVIP and TMT-B are thought to have a verbal WM component (Coull et al., 1996; Sánchez-Cubillo et al., 2009), our results could underpin the hypothesis of an association between verbal WM and successful AS performance (Mitchell et al., 2002; Roberts et al., 1994; Unsworth et al., 2004). In our study, a test measuring verbal WM span, similar to Digit Span or OS-SPAN, was missing and as a consequence we cannot draw reliable conclusions about whether and to what extent successful AS performance demands adequate verbal WM. It would, therefore, be interesting to further study the possible associations of verbal WM with AS performance with a wider range of tests that measure verbal WM more directly. Further studies should also strive to add to our understanding of verbal WM and goal activation, how these factors associate and affect AS performance, as studies have indicated that goal activation partly explains the association between verbal WM and AS performance (Hutton, 2008; Kane et al., 2001; Meier et al., 2017).

Some models have suggested that response inhibition is a separate and critical factor for successful AS performance, assuming that it is necessary to cancel the automatic prosaccade response for the AS response to be evoked (Everling & Fischer, 1998; Guitton et al., 1985; Munoz & Everling, 2004; Noorani & Carpenter, 2013; Roberts et al., 1994). However, the importance of inhibition was shown not to be as strong in our research, because neither the number of perseverative errors on the WCST nor the reaction time when naming colors on the Stroop task significantly predicted the outcome on the AS task. This conclusion of course has to be tempered by the task impurity of our inhibitory measures in this study, but is generally in line with several other studies that have not found such associations (Barton et al., 2002; Snitz et al., 1999). As such, these findings support our hypothesis of attention and WM components being of particular importance for AS performance. Other studies have similarly shown only very low and sometimes non-significant correlations between AS error rate and other measures of inhibitory control (Aichert et al., 2012; Miyake et al., 2000; Rey-Mermet et al., 2018; Stahl et al., 2014).

A notable feature of this study is that it is larger than some previous studies on healthy populations, both with regards to the number of participants and applied tests, which adds weight to the results. Furthermore, all subjects were tested at the same research center by psychologists that received the same standardized training and their assessments were regularly monitored by a supervisor to ensure consistency. Moreover, the sample was not drawn from a university student sample with restricted range in cognitive ability, as is frequently done in other studies. The sample of our study was thoroughly screened with regard to drug use as well as neurological and psychiatric conditions known to affect performance on both cognitive and AS tasks. Therefore, whilst our sample has the advantage of representing a larger segment of the general population than student samples, it can be expected that the cognitive function levels of our sample varied less than in more heterogeneous, unscreened samples.

However, a limitation of our study is that, despite the wide range of cognitive tests that were used, further tests on both WM and attention would have been useful to draw more definite conclusions. All of the tests used here measure more than one cognitive process, a well-known problem in psychology (Miyake et al., 2000) which admittedly complicates the interpretation of the results. Further studies using different tests could possibly find that other cognitive factors are better predictors of AS performance; therefore, it is important to continue studying the cognitive mechanisms of AS performance. Additionally, the current study used a fairly large age range. Whilst this represents a strength, as we were aiming not to restrict our sample to young participants as in studies of university students, a potential limitation of this design is that different patterns of antisaccade correlates may occur in younger than in older adults, an effect that may obscure findings in the entire sample. Future research in sufficiently powered samples of young and older participants are needed to satisfactorily address this issue. Finally, it is also important to take into account that although TMT-B and RVIP were statistically significant predictors of AS performance, they only explained a small proportion of the variance, with RVIP explaining 8.5% of the variance of AS error rate and TMT-B explaining 9.7% of the variance of AS latency. Therefore, there is still room for other factors to account for the large variance in AS performance among healthy individuals.

In conclusion, the results of our exploratory study indicate that TMT-B predict predicts AS latency while RVIP was the best predictor of error rate on the AS task. As both tasks measure a range of cognitive functions including WM, attention and flexibility, the results need to be interpreted in the light of earlier research with the aim of adding to our understanding of the cognitive mechanisms underlying AS performance. Further association studies on healthy populations, using a wider range of attention and specific WM tests, would be useful to further our understanding of the cognitive control of AS performance.

## Data Availability

The data from the study are part of a larger study by deCODE genetics. Although the company only analyses encrypted data its policy is not to share individual-level data.

## Footnotes

Introductions to Bayesian statistics are available e.g. in McElreath (2016). How Bayesian statistics can be used to investigate “null-effects” is covered in Harms & Lakens (2018).

## References

[1] Aichert, D. S., Wöstmann, N. M., Costa, A., Macare, C., Wenig, J. R., Möller, H.-J., Ettinger, U., et al. (2012). Associations between trait impulsivity and prepotent response inhibition. Journal of Clinical and Experimental Neuropsychology, 34(10), 1016–1032. DOI: 10.1080/13803395.2012.706261 22888795

[2] Barton, J. J. S., Cherkasova, M. V., Lindgren, K., Goff, D. C., Intriligator, J. M., & Manoach, D. S. (2002). Antisaccades and Task Switching. Annals of the New York Academy of Sciences, 956(1), 250–263. DOI: 10.1111/j.1749-6632.2002.tb02824.x 11960809

[3] Benton, A. H. K. (1989). Multilingual Aphasia Examination. AJA Associates.

[4] Berg, E. A. (1948). A Simple Objective Technique for Measuring Flexibility in Thinking. The Journal of General Psychology, 39(1), 15–22. DOI: 10.1080/00221309.1948.9918159 18889466

[5] Chuderski, A. (2014). How well can storage capacity, executive control, and fluid reasoning explain insight problem solving. Intelligence, 46, 258–270. DOI: 10.1016/j.intell.2014.07.010

[6] Clyde, M. (2018). BAS: Bayesian Variable Selection and Model Averaging using Bayesian Adaptive Sampling, R package, Version 1.5.3. Retrieved from: https://cran.r-project.org/web/packages/BAS/BAS.pdf.

[7] Connolly, J. D., Goodale, M. A., Goltz, H. C., & Munoz, D. P. (2005). fMRI Activation in the Human Frontal Eye Field Is Correlated With Saccadic Reaction Time. Journal of Neurophysiology, 94(1), 605–611. DOI: 10.1152/jn.00830.2004 15590732

[8] Connolly, J. D., Goodale, M. A., Menon, R. S., & Munoz, D. P. (2002). Human fMRI evidence for the neural correlates of preparatory set. Nature Neuroscience, 5(12), 1345–1352. DOI: 10.1038/nn969 12411958

[9] Coull, J. T., Frith, C. D., Frackowiak, R. S. J., & Grasby, P. M. (1996). A fronto-parietal network for rapid visual information processing: A PET study of sustained attention and working memory. Neuropsychologia, 34(11), 1085–1095. DOI: 10.1016/0028-3932(96)00029-2 8904746

[10] Crawford, T. J., Bennett, D., Lekwuwa, G., Shaunak, S., & Deakin, J. F. W. (2002). Cognition and the inhibitory control of saccades in schizophrenia and Parkinson’s disease In: Progress in Brain Research, 140, 449–466. Elsevier DOI: 10.1016/S0079-6123(02)40068-4 12508608

[11] Cutsuridis, V., Smyrnis, N., Evdokimidis, I., & Perantonis, S. (2007). A neural model of decision-making by the superior colicullus in an antisaccade task. Neural Networks, 20(6), 690–704. DOI: 10.1016/j.neunet.2007.01.004 17446043

[12] Ettinger, U., Ffytche, D. H., Kumari, V., Kathmann, N., Reuter, B., Zelaya, F., & Williams, S. C. R. (2008). Decomposing the Neural Correlates of Antisaccade Eye Movements Using Event-Related fMRI. Cerebral Cortex, 18(5), 1148–1159. DOI: 10.1093/cercor/bhm147 17728263

[13] Everling, S., & Fischer, B. (1998). The antisaccade: A review of basic research and clinical studies. Neuropsychologia, 36(9), 885–899. DOI: 10.1016/S0028-3932(98)00020-7 9740362

[14] Feigenbaum, J. D., Polkey, C. E., & Morris, R. G. (1996). Deficits in spatial working memory after unilateral temporal lobectomy in man. Neuropsychologia, 34(3), 163–176. DOI: 10.1016/0028-3932(95)00107-7 8868274

[15] Gau, S. S.-F., & Huang, W.-L. (2014). Rapid visual information processing as a cognitive endophenotype of attention deficit hyperactivity disorder. Psychological Medicine, 44(02), 435–446. DOI: 10.1017/S0033291713000640 23561037

[16] Golden, C. J., & Freshwater, S. M. (1978). The Stroop Color and Word Test: A Manual for Clinical and Experimental Uses. Chicago: Stoelting.

[17] Gudmundsson, E. (2016). WASI-IS Wechsler Abbreviated Scale of Intelligence Icelandic Standardisation. Reykjavik: Menntamalastofnun.

[18] Guitton, D., Buchtel, H. A., & Douglas, R. M. (1985). Frontal lobe lesions in man cause difficulties in suppressing reflexive glances and in generating goal-directed saccades. Experimental Brain Research, 58(3). DOI: 10.1007/BF00235863 4007089

[19] Hallett, P. E. (1978). Primary and secondary saccades to goals defined by instructions. Vision Research, 18(10), 1279–1296. DOI: 10.1016/0042-6989(78)90218-3 726270

[20] Heaton, R., & Staff, A. R. (1993). “Wisconsin card sorting test: Computer version-2”. Odesssa: Psychological Assessment Resources.

[21] Hoeting, J. A., Madigan, D., Raftery, A. E., & Volinsky, C. T. (1999). Bayesian model averaging: A tutorial. Statistical science, 382–401.

[22] Hutton, S. B. (2008). Cognitive control of saccadic eye movements. Brain and Cognition, 68(3), 327–340. DOI: 10.1016/j.bandc.2008.08.021 19028265

[23] Hutton, S. B., & Ettinger, U. (2006). The antisaccade task as a research tool in psychopathology: A critical review. Psychophysiology, 43(3), 302–313. DOI: 10.1111/j.1469-8986.2006.00403.x 16805870

[24] Kane, M. J., Bleckley, M. K., Conway, A. R. A., & Engle, R. W. (2001). A controlled-attention view of working-memory capacity. Journal of Experimental Psychology: General, 130(2), 169–183. DOI: 10.1037/0096-3445.130.2.169 11409097

[25] Kristjansson, A. (2007). Saccade landing point selection and the competition account of pro- and antisaccade generation: The involvement of visual attention? A review. Scandinavian Journal of Psychology, 48(2), 97–113. DOI: 10.1111/j.1467-9450.2007.00537.x 17430363

[26] Kristjansson, A., Chen, Y., & Nakayama, K. (2001). Less attention is more in the preparation of antisaccades, but not prosaccades. Nature Neuroscience, 4(10), 1037–1042. DOI: 10.1038/nn723 11547337

[27] Kristjansson, A., Vandenbroucke, M. W. G., & Driver, J. (2004). When pros become cons for anti-versus prosaccades: Factors with opposite or common effects on different saccade types. Experimental Brain Research, 155(2), 231–244. DOI: 10.1007/s00221-003-1717-9 14661119

[28] Levy, D. L., Mendell, N. R., & Holzman, P. S. (2004). The antisaccade task and neuropsychological tests of prefrontal cortical integrity in schizophrenia: Empirical findings and interpretative considerations. World Psychiatry, 3(1), 32. 16633452 PMC1414662

[29] Lezak, M. D., Howieson, D. B., & Loring, D. W. (2012). Neuropsychological Assessment (5th ed.). New York: Oxford University Press.

[30] Massen, C. (2004). Parallel programming of exogenous and endogenous components in the antisaccade task. The Quarterly Journal of Experimental Psychology Section A, 57(3), 475–498. DOI: 10.1080/02724980343000341 15204137

[31] McDowell, J. E., & Clementz, B. (2001). Behavioral and brain imaging studies of saccadic performance in schizophrenia. Biological Psychology, 57(1–3), 5–22. DOI: 10.1016/S0301-0511(01)00087-4 11454432

[32] McDowell, J. E., Kissler, J. M., Berg, P., Dyckman, K. A., Gao, Y., Rockstroh, B., & Clementz, B. A. (2005). Electroencephalography/magnetoencephalography study of cortical activities preceding prosaccades and antisaccades. Neuroreport, 16(7), 663–668. DOI: 10.1097/00001756-200505120-00002 15858402

[33] Meier, M. E., Smeekens, B. A., Silvia, P. J., Kwapil, T. R., & Kane, M. J. (2017). Working Memory Capacity and the Antisaccade Task: A Microanalytic-Macroanalytic Investigation of Individual Differences in Goal Activation and Maintenance. Journal of Experimental Psychology. Learning, Memory, and Cognition. DOI: 10.1037/xlm0000431 PMC5741546 28639800

[34] Mitchell, J. P., Macrae, C. N., & Gilchrist, I. D. (2002). Working Memory and the Suppression of Reflexive Saccades. Journal of Cognitive Neuroscience, 14(1), 95–103. DOI: 10.1162/089892902317205357 11798390

[35] Miyake, A., Friedman, N. P., Emerson, M. J., Witzki, A. H., Howerter, A., & Wager, T. D. (2000). The Unity and Diversity of Executive Functions and Their Contributions to Complex “Frontal Lobe” Tasks: A Latent Variable Analysis. Cognitive Psychology, 41(1), 49–100. DOI: 10.1006/cogp.1999.0734 10945922

[36] Moon, S. Y., Barton, J. J. S., Mikulski, S., Polli, F. E., Cain, M. S., Vangel, M., Manoach, D. S., et al. (2007). Where left becomes right: A magnetoencephalographic study of sensorimotor transformation for antisaccades. NeuroImage, 36(4), 1313–1323. DOI: 10.1016/j.neuroimage.2007.04.040 17537647 PMC1995561

[37] Munakata, Y., Herd, S. A., Chatham, C. H., Depue, B. E., Banich, M. T., & O’Reilly, R. C. (2011). A unified framework for inhibitory control. Trends in Cognitive Sciences, 15(10), 453–459. DOI: 10.1016/j.tics.2011.07.011 21889391 PMC3189388

[38] Munoz, D. P., & Everling, S. (2004). Look away: The anti-saccade task and the voluntary control of eye movement. Nature Reviews Neuroscience, 5(3), 218–228. DOI: 10.1038/nrn1345 14976521

[39] Noorani, I., & Carpenter, R. H. S. (2013). Antisaccades as decisions: LATER model predicts latency distributions and error responses. European Journal of Neuroscience, 37(2), 330–338. DOI: 10.1111/ejn.12025 23121177

[40] Owen, A. M., Downes, J. J., Sahakian, B. J., Polkey, C. E., & Robbins, T. W. (1990). Planning and spatial working memory following frontal lobe lesions in man. Neuropsychologia, 28(10), 1021–1034. DOI: 10.1016/0028-3932(90)90137-D 2267054

[41] Pagnoni, G. (2012). Dynamical Properties of BOLD Activity from the Ventral Posteromedial Cortex Associated with Meditation and Attentional Skills. Journal of Neuroscience, 32(15), 5242–5249. DOI: 10.1523/JNEUROSCI.4135-11.2012 22496570 PMC3362741

[42] Pierrot-Deseilligny, C., Ploner, C. J., MüRi, R. M., Gaymard, B., & Rivaud-PéChoux, S. (2002). Effects of Cortical Lesions on Saccadic. Annals of the New York Academy of Sciences, 956(1), 216–229. DOI: 10.1111/j.1749-6632.2002.tb02821.x 11960806

[43] Ploner, C. J., Gaymard, B. M., Rivaud-Péchoux, S., & Pierrot-Deseilligny, C. (2005). The prefrontal substrate of reflexive saccade inhibition in humans. Biological Psychiatry, 57(10), 1159–1165. DOI: 10.1016/j.biopsych.2005.02.017 15866556

[44] Radant, A. D., Claypoole, K., Wingerson, D. K., Cowley, D. S., & Roy-Byrne, P. P. (1997). Relationships between Neuropsychological and Oculomotor Measures in Schizophrenia Patients and Normal Controls. Biological Psychiatry, 42(9), 797–805. DOI: 10.1016/S0006-3223(96)00464-7 9347128

[45] Raftery, A. E., Madigan, D., & Hoeting, J. A. (1997). Bayesian model averaging for linear regression models. Journal of the American Statistical Association, 92(437), 179–191. DOI: 10.1080/01621459.1997.10473615

[46] Reitan, R. M. (1958). Validity of the Trail Making Test as an Indicator of Organic Brain Damage. Perceptual and Motor Skills, 8(3), 271–276. DOI: 10.2466/pms.1958.8.3.271

[47] Reitan, R. M., & Wolfson, D. (1985). The Halstead-Reitan Neuropsychological Test Battery: Therapy and clinical interpretation. Tucson, AZ: Neuropsychological Press.

[48] Reuter, B., & Kathmann, N. (2004). Using saccade tasks as a tool to analyze executive dysfunctions in schizophrenia. Acta Psychologica, 115(2–3), 255–269. DOI: 10.1016/j.actpsy.2003.12.009 14962403

[49] Reuter, B., Rakusan, L., & Kathmanna, N. (2005). Poor antisaccade performance in schizophrenia: An inhibition deficit? Psychiatry Research, 135(1), 1–10. DOI: 10.1016/j.psychres.2004.12.006 15893384

[50] Rey-Mermet, A., Gade, M., & Oberauer, K. (2018). Should we stop thinking about inhibition? Searching for individual and age differences in inhibition ability. Journal of Experimental Psychology: Learning, Memory, and Cognition, 44(4), 501–526. DOI: 10.1037/xlm0000450 28956944

[51] Roberts, R. J., Hager, L. D., & Heron, C. (1994). Prefrontal cognitive processes: Working memory and inhibition in the antisaccade task. Journal of Experimental Psychology: General, 123(4), 374–393. DOI: 10.1037/0096-3445.123.4.374

[52] Sahakian, B. J., & Owen, A. M. (1992). Computerized assessment in neuropsychiatry using CANTAB: Discussion paper. Journal of the Royal Society of Medicine, 85(7), 399–402. 1629849 PMC1293547

[53] Sánchez-Cubillo, I., Periáñez, J. A., Adrover-Roig, D., Rodríguez-Sánchez, J. M., Ríos-Lago, M., Tirapu, J., & Barceló, F. (2009). Construct validity of the Trail Making Test: Role of task-switching, working memory, inhibition/interference control, and visuomotor abilities. Journal of the International Neuropsychological Society, 15(3), 438–450. DOI: 10.1017/S1355617709090626 19402930

[54] Scarpina, F., & Tagini, S. (2017). The Stroop Color and Word Test. Frontiers in Psychology, 8 DOI: 10.3389/fpsyg.2017.00557 PMC5388755 28446889

[55] Shipstead, Z., Harrison, T. L., & Engle, R. W. (2015). Working memory capacity and the scope and control of attention. Attention, Perception, & Psychophysics, 77(6), 1863–1880. DOI: 10.3758/s13414-015-0899-0 25911154

[56] Snitz, B. E., Curtis, C. E., Zald, D. H., Katsanis, J., & Iacono, W. G. (1999). Neuropsychological and oculomotor correlates of spatial working memory performance in schizophrenia patients and controls. Schizophrenia Research, 38(1), 37–50. DOI: 10.1016/S0920-9964(98)00178-9 10427609

[57] Stahl, C., Voss, A., Schmitz, F., Nuszbaum, M., Tüscher, O., Lieb, K., & Klauer, K. C. (2014). Behavioral components of impulsivity. Journal of Experimental Psychology: General, 143(2), 850–886. DOI: 10.1037/a0033981 23957282

[58] Stefansson, H., Meyer-Lindenberg, A., Steinberg, S., Magnusdottir, B., Morgen, K., Arnarsdottir, S., Stefansson, K., et al (2014). CNVs conferring risk of autism or schizophrenia affect cognition in controls. Nature, 505(7483), 361–366. DOI: 10.1038/nature12818 24352232

[59] Strauss, E., Sherman, E. M. S., & Spreen, O. (2006). A Compendium of Neuropsychological Tests: Administration, Norms, and Commentary. Oxford University Press.

[60] Stroop, J. R. (1935). Studies of interference in serial verbal reactions. Journal of Experimental Psychology, 18(6), 643–662. DOI: 10.1037/h0054651

[61] Unsworth, N., & Engle, R. W. (2007). The nature of individual differences in working memory capacity: Active maintenance in primary memory and controlled search from secondary memory. Psychological Review, 114(1), 104–132. DOI: 10.1037/0033-295X.114.1.104 17227183

[62] Unsworth, N., Schrock, J. C., & Engle, R. W. (2004). Working Memory Capacity and the Antisaccade Task: Individual Differences in Voluntary Saccade Control. Journal of Experimental Psychology: Learning, Memory, and Cognition, 30(6), 1302–1321. DOI: 10.1037/0278-7393.30.6.1302 15521806

[63] Unsworth, N., & Spillers, G. J. (2010). Working memory capacity: Attention control, secondary memory, or both? A direct test of the dual-component model. Journal of Memory and Language, 62(4), 392–406. DOI: 10.1016/j.jml.2010.02.001

[64] Van der Elst, W., Van Boxtel, M. P. J., Van Breukelen, G. J. P., & Jolles, J. (2006). The Stroop Color-Word Test: Influence of Age, Sex, and Education; and Normative Data for a Large Sample Across the Adult Age Range. Assessment, 13(1), 62–79. DOI: 10.1177/1073191105283427 16443719

[65] Wang, D., Zhang, W., & Bakhai, A. (2004). Comparison of Bayesian model averaging and stepwise methods for model selection in logistic regression. Statistics in medicine, 23(22), 3451–3467. DOI: 10.1002/sim.1930 15505893

[66] Wasserman, L. (2000). Bayesian model selection and model averaging. Journal of mathematical psychology, 44(1), 92–107. DOI: 10.1006/jmps.1999.1278 10733859

[67] Weber, H., Dürr, N., & Fischer, B. (1998). Effects of pre-cues on voluntary and reflexive saccade generation. Experimental Brain Research, 120(4), 417–431. DOI: 10.1007/s002210050415 9655227

[68] Zhou, X., Zhu, D., King, S. G., Lees, C. J., Bennett, A. J., Salinas, E., Constantinidis, C., et al. (2016). Behavioral response inhibition and maturation of goal representation in prefrontal cortex after puberty. Proceedings of the National Academy of Sciences of the United States of America, 113(12), 3353–3358. DOI: 10.1073/pnas.1518147113 26951656 PMC4812761

